# Potential of Natural Alkaloids From Jadwar (*Delphinium denudatum*) as Inhibitors Against Main Protease of COVID-19: A Molecular Modeling Approach

**DOI:** 10.3389/fmolb.2022.898874

**Published:** 2022-05-10

**Authors:** Anuj Kumar, Mansi Sharma, Christopher D. Richardson, David J. Kelvin

**Affiliations:** ^1^ Laboratory of Immunity, Shantou University Medical College, Shantou, China; ^2^ Department of Microbiology and Immunology, Canadian Centre for Vaccinology CCfV, Faculty of Medicine, Dalhousie University, Halifax, Canada

**Keywords:** COVID-19, alkaloids, Jadwar, molecular docking, molecular dynamics simulations and free energy

## Abstract

The ongoing pandemic coronavirus disease (COVID-19) caused by a novel corona virus, namely, severe acute respiratory syndrome coronavirus-2 (SARS-CoV-2), has had a major impact on global public health. COVID-19 cases continue to increase across the globe with high mortality rates in immunocompromised patients. There is still a pressing demand for drug discovery and vaccine development against this highly contagious disease. To design and develop antiviral drugs against COVID-19, the main protease (M^pro^) has emerged as one of the important drug targets. In this context, the present work explored Jadwar (*Delphinium denudatum*)–derived natural alkaloids as potential inhibitors against M^pro^ of SARS-CoV-2 by employing a combination of molecular docking and molecular dynamic simulation–based methods. Molecular docking and interaction profile analysis revealed strong binding on the M^pro^ functional domain with four natural alkaloids *viz*. panicutine (−7.4 kcal/mol), vilmorrianone (−7.0 kcal/mol), denudatine (−6.0 kcal/mol), and condelphine (−5.9 kcal/mol). The molecular docking results evaluated by using the MD simulations on 200 nanoseconds confirmed highly stable interactions of these compounds with the M^pro^. Additionally, mechanics/generalized Born/Poisson–Boltzmann surface area (MM/G/P/BSA) free energy calculations also affirmed the docking results. Natural alkaloids explored in the present study possess the essential drug-likeness properties, namely, absorption, distribution, metabolism, and excretion (ADME), and are in accordance with Lipinski’s rule of five. The results of this study suggest that these four bioactive molecules, namely, condelphine, denudatine, panicutine, and vilmorrianone, might be effective candidates against COVID-19 and can be further investigated using a number of experimental methods.

## Introduction

The unprecedented pandemic of coronavirus disease (COVID-19) was caused by a novel coronavirus, namely, severe acute respiratory syndrome coronavirus-2 (SARS-CoV-2), which appeared in Wuhan, Hubei Province, China, at the end of December 2019 (Jamwal et al., 2020). A few months later, this viral disease spread to 219 nations and territories across the globe. On 30 January 2020, the World Health Organization (WHO) declared this contagious disease as a Public Health Emergency of International Concern (PHEIC) and based on the recommendations of the Emergency Committee announced it to be a pandemic on 11 March 2020 (Shi et al., 2020; Wilder-Smith and Osman 2020; Yu and Yu 2020). The ongoing pandemic eruption adversely affected the global economy and financial markets (Pak et al., 2020). As of 3 March 2022, COVID-19 has led to more than 438,968,263 confirmed cases and 5,969,439 deaths (https://covid19.who.int/), with high mortality rates in immunocompromised and elderly patients. A large number of Canadians were infected with SARS-CoV-2 during the different waves of the ongoing pandemic. As of 3 March 2022, the total caseload in Canada has soared to 3,296,503 with 36,638 fatalities (https://covid19.who.int/region/amro/country/ca). Based on infection, morbidity, and mortality, this respiratory infectious disease has greatly superseded previous outbreaks of SARS and the Middle East respiratory syndrome (MERS) (de Wit et al., 2016; Wang et al., 2020; Yuan et al., 2020). Previous SARS and MERS outbreaks possessed fatality rates of 10 and 35%, respectively (Lee et al., 2004; Cheng et al., 2007). It has been reported that COVID-19 is associated with disorders in the respiratory and digestive tracts of the body (Chen et al., 2020; Pal et al., 2020; Tang et al., 2020).

Morphologically, the coronaviruses (CoVs) are a highly diverse family of enveloped positive-sense single-strand RNA viruses (Fehr and Perlman 2015). The Coronavirinae are classified into two subfamilies: Coronavirinae and Torovirinae. Based on the molecular structure and biological functions, the Coronavirinae are further divided into four genera: alpha- (α-), beta- (β-), gamma- (γ-), and delta-coronavirus (δ-CoV) (Hulswit et al., 2016; Payne 2017). To date, six human coronavirus species have been identified, namely, HCoV-NL63, HCoV-229E, HCoV-OC34, HCoV-HKU1, SARS-CoV, and MERS-CoV (Arden et al., 2005; Su et al., 2016; Zhang et al., 2018). The novel strain SARS-CoV-2 has been reported as the seventh CoV known to infect humans (Andersen et al., 2020) in the genus *Betacoronavirus* (https://talk.ictvonline.org/; Helmy et al., 2020; Wang et al., 2020).

The single-stranded positive RNA genome of the SARS-CoV-2 virus is ∼29.9 kb in size (Wu et al., 2020). The genome sequence for the Wuhan-Hu-1 strain of SARS-CoV-2 is available from the GenBank with the accession number MN908947 (∼29,903 nucleotides) (Wu et al., 2020). The SARS-CoV-2 genome contains 14 open reading frames (ORFs) encoding 27 proteins (Alsobaie 2021). The 5′ untranslated region (UTR) end harbors ORF1a/b that produces a polyprotein which is posttranslationally cleaved into 16 different nonstructural proteins (nsp1–nsp16). These form the replicase/transcriptase complex (RTC). They include a papain-like protease (nsp3), main protease (M^pro^, 3CL^pro^, nsp5), nsp7–nsp8 primase complex, primary RNA-dependent RNA polymerase (RdRp; nsp12), helicase/triphosphatase (nsp13), exoribonuclease (nsp14), endonuclease (nsp15), and N7- and 2′-O-methyltransferases (nsp10/nsp16). The 3′-end of the SARS-CoV-2 contains ORFs which encode the four structural proteins, namely, E (envelope protein), M (membrane protein), N (nucleocapsid protein), and S (spike protein), as well as nine putative accessory factors (Rastogi et al., 2020; Alsobaie 2021; Shamsi et al., 2021). The SARS-CoV-2 M^pro^ (nsp5) is encoded by the major ORF1ab following posttranslational cleavage in the cytosol (Ullrich and Nitsche 2020; Mengist et al., 2021). Based on its key role in mediating viral replication and transcription, M^pro^ has been considered as one of the promising drug targets against SARS-CoV-2 (Jin et al., 2020; Ullrich and Nitsche 2020; Mengist et al., 2021). M^pro^ consists of 306 amino acids yielding a molecular mass of 33,797 Da (Khan et al., 2020). M^pro^ is composed of three functional domains: domain I (8-101 aa), domain II (102-184 aa), and domain III (201-306 aa) (Jin et al., 2020; Kumar et al., 2021). Based on structure topology, it has been well reported that an antiparallel *ß*-barrel structure is present in domains I and II. While domain III was found to possess a set of five α-helices organized as a large antiparallel cluster. Domains II and III were connected to each other with the help of a 15-residue-long loop region (185–200 residues). Numerous *in vitro*, *in vivo*, and *in silico* studies have been performed to screen the candidate natural compounds as potential inhibitors of SARS-CoV M^pro^ (Benarba and Pandiella 2020; Chikhale et al., 2020; Joshi et al., 2020; Kumar et al., 2020; Raj et al., 2020; Sharma et al., 2020; Tripathi et al., 2020; Yadav et al., 2020; Chowdhury 2021; Mishra et al., 2021; Mukherjee et al., 2021; Patel et al., 2021; Rangsinth et al., 2021; Teli et al., 2021). By now, many FDA-approved known inhibitors of viral protease such as HIV-1 (atazanavir, amprenavir, darunavir, nelfinavir, tipranavir, lopinavir, saquinavir, indinavir, and ritonavir) and hepatitis C virus (ritonavir, boceprevir, telaprevir, paritaprevir, asunaprevir, grazoprevir, glecaprevir, voxilaprevir, and sofosbuvir) have been proposed for the treatment of COVID-19 (Lv et al., 2015; de Leuw and Stephan 2017; Abdelli et al., 2020; Das et al., 2020; Khan et al., 2020; and Mengist et al., 2021). Chloroquine, an FDA-approved antimalarial drug has also been explored as a potential inhibitor of M^pro^ (Ou et al., 2021). In a recent study, two drugs, namely, rifampicin and letermovir have been repurposed as inhibitors of M^pro^ based on investigations of their molecular docking (Pathak et al., 2021). So far, no effective method has been developed for the treatment of this contagious disease, therefore, there is an urgent need to design targeted therapeutic agents to prevent and treat COVID-19.

*Delphinium denudatum* Wall (Ranunculaceae), also known as Jadwar, is an annual or perennial ornamental shrub that grows to a height of 40–80 cm. This plant grows at high altitudes ranging from 2,400 to 36,500 m on the outer ranges of the western Himalayas from Kashmir to Uttarakhand (Nizami and Jafri 2006; Kumar et al., 2018; Singh et al., 2018). Different portions of *D. denudatum* have been used medicinally for centuries (Pelletier 1996; Nizami and Jafri 2006). The extracts of Jadwar have been shown to exhibit neuroprotective and cardioprotective properties (Kumar et al., 2018; Singh KP, Kumar A, 2018). Its roots have many diverse uses such as analgesic, antipyretic, antiseptic, anti-inflammatory, aphrodisiac, antidote (for the snake’s venom), cardiotonic, diuretic, exhilarant, sedative, and solvent applications (Abid et al., 2017; Singh KP, Kumar A, 2018). It is well documented that Jadwar is traditionally used for the treatment of various diseases such as fungal infections, cardiac diseases, cholera, epilepsy, jaundice, mania, migraine, paralysis, pain, snake bite, scorpion sting, toothache, etc. (Atta-ur-Rahman et al., 1997; Atta-ur-Rahman and Choudhary 1999; Raza et al., 2003; Ahmad et al., 2017; Kumar et al., 2018). Also, the Jadwar root is used in morphine de-addiction therapy (Zafar et al., 2001; Rahman et al., 2002). A plethora of natural compounds belong to flavonoids, triterpenoids, and alkaloids, such as delphocurarine, staphisagrine, delphine, condelphine, and denudatin. A diterpenoid alkaloid identical to condelphine is exclusively found in Jadwar (Singh and Chopra 1962; Jain et al., 2021). Jadwar-derived isotalatazidine hydrate has demonstrated cholinesterase inhibitory potential and can be used as the target drug for Alzheimer's disease (Ahmad et al., 2017). Despite the rich pharmacological properties of Jadwar, natural compounds exclusively found in this important medicinal herb have not yet been explored for the treatment of SARS-CoV-2. Available open-source platforms and molecular modeling algorithms can be utilized to explore the potential of Jadwar-derived natural alkaloids as potential inhibitors against targets of COVID-19.

In the present study, we have employed a molecular modeling approach to assess the potential of Jadwar-derived natural alkaloids (panicutine, vilmorrianone, denudatine, and condelphine) as inhibitors of M^pro^ from SARS-CoV-2. These bioactive compounds were subjected to molecular docking analysis with M^pro^ enzyme. Docking complexes were further evaluated for conformational stability using molecular dynamics simulations of 200 ns followed by mechanics/generalized Born/Poisson–Boltzmann surface area (MM/G/P/BSA) free energy calculations.

## Materials and Methods

**FIGURE 1:**
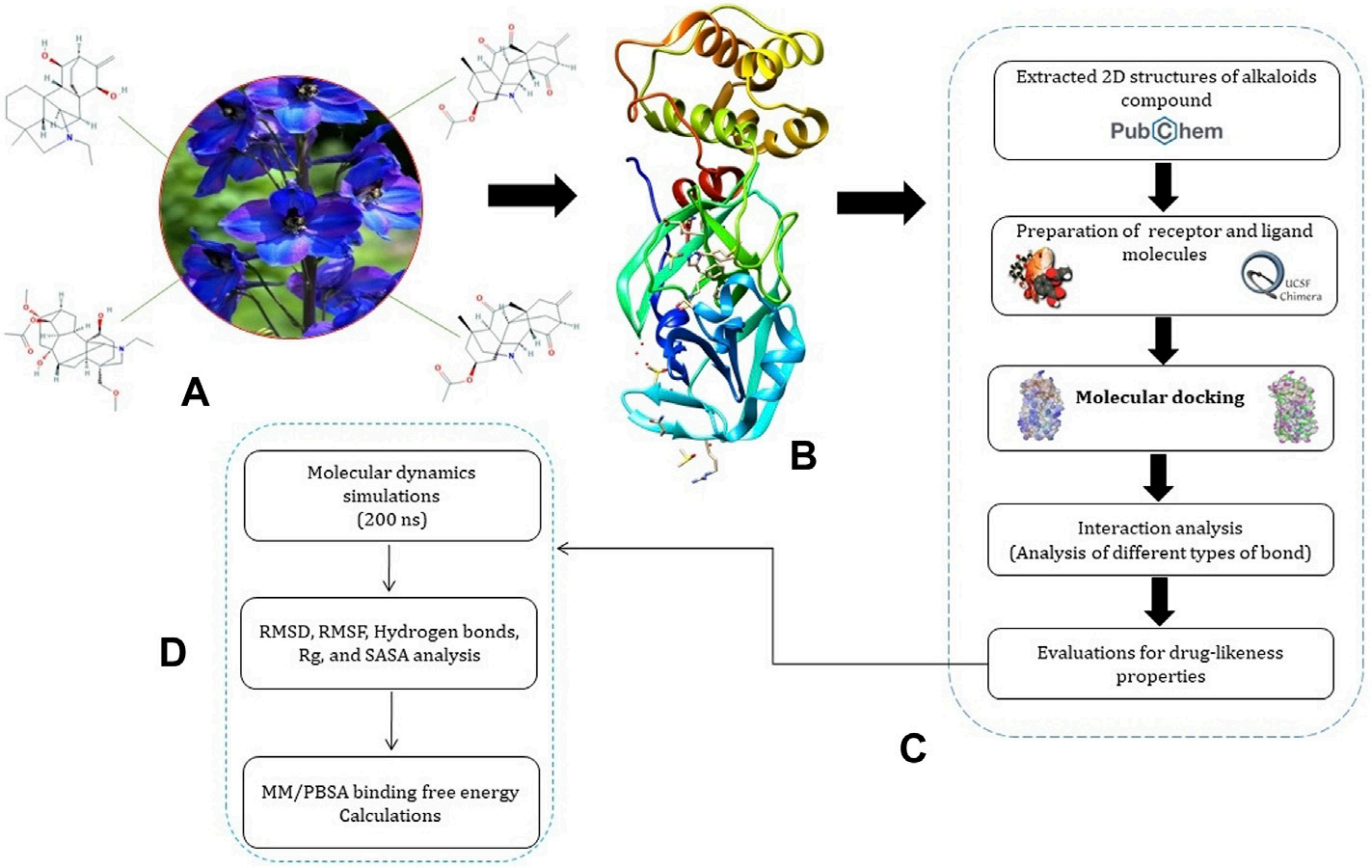
Representation of pipeline. **(A)** 2D structures of Jadwar-derived natural alkaloids (condelphine, denudatine, panicutine, and vilmorrianone) utilized in the present study; **(B)** 3D structure of M^pro^ (PDB ID: 5R7Y) of COVID-19 obtained from the RCSB-PDB; **(C)** steps involved in the preparation of receptor and ligand molecules followed by docking; and **(D)** flow of steps utilized for performing molecular dynamics simulations and binding free energy calculations on 200 ns.

A systematic approach of the molecular modeling used in this study is presented in Figure 1.

### Protein Structure Retrieval and Preparation

We retrieved the crystal structure of SARS-CoV-2 M^pro^ in complex with an inhibitor Z45617795 (PDB ID: 5R7Y, at a resolution of 1.65 Å, R-Value Free of 0.237, and R-Value Work of 0.179) from the RCSB Protein Data Bank (RCSB-PDB) (https://www.rcsb.org/) in PDB format (Berman 2000; Burley et al., 2018). The protein structure was prepared using AutoDockTools version 1.5.6, UCSF Chimera, and Discovery Studio. Prior to molecular docking, water and hetero atoms were removed, polar hydrogen was added, and Kollman charges were assigned as well on the receptor protein. Amber force field ff14SB embedded in UCSF Chimera was applied for protein structure optimization and energy minimization. The side chain correction was executed using the clean geometry algorithm monitored in the Discovery Studio platform.

### Ligand Structure Retrieval and Preparation

In a search for a potential drug candidate against COVID-19, the four Jadwar-derived natural alkaloids, namely, panicutine, vilmorrianone, denudatine, and condelphine, that have been reported to have therapeutic potential were selected based on an extensive literature survey. The comprehensive PubChem repository was utilized to extract the 2D structures of these alkaloids—denudatine (CID_161515), condelphine (CID_441720), vilmorrianone (CID_44566629), and panicutine (CID_44566630)—in the SDF format (Kim et al., 2020). 3D and geometry optimizations with energy minimization for each molecule were performed using the UCSF Chimera program. The Open Babel toolbox (O’Boyle et al., 2011) which is available in the PyRx package was utilized to convert these molecules into the PDBQT format. All four compounds were prepared by adding the polar hydrogens and Gasteiger charges as previously described in Kumar et al. (2021).

### Molecular Docking

To predict the molecular interactions between M^pro^ of SARS-CoV-2 and the four natural alkaloid compounds—denudatine, condelphine, vilmorrianone, and panicutine—we have performed molecular docking to identify the interaction between the chemical molecules and target proteins. Molecular docking was done with AutoDock v4.2 (Morris et al., 2009), and the binding affinity score was calculated for the docking complexes. Eleven amino acid residues, namely, Thr24, Thr26, Asn119, Phe140, Gly143, Cys145, His163, His164, Glu166, Gln189, and Thr190, were used as the active sites of the receptor protein. These active site residues were considered based on the previous reports by Khan et al. (2020) and Kumar et al. (2021). During the molecular docking process, the M^pro^ was fixed, while the ligand molecules were flexible. A grid box was created with dimensions 60 Å × 60 Å × 60 Å centered at the coordinates X = −10, Y = 13, and Z = 70, with 100 conformations for each molecule based on the Lamarckian genetic algorithm (LGA) (Fuhrmann et al., 2010). The representative binding position for the ligand molecules was selected based on the negative binding energy and binding interactions with the receptor protein.

### Drug Likeness Properties

In terms of absorption, distribution, metabolism, and excretion, the ADME and drug-likeness of all four alkaloid compounds were predicted using the SWISS-ADME server (Daina et al., 2017). During the drug-likeness prediction process, all four alkaloids were analyzed based on the Lipinski’s rule of five (Lipinski 2004), using Veber’s rule (Veber et al., 2002), polar surface area (TPSA), bioavailability, and solubility potential (Daina et al., 2017).

### Molecular Dynamics Simulations

To elucidate the behavior of the natural alkaloids (panicutine, vilmorrianone, denudatine, and condelphine) binding to the M^pro^ of COVID-19 and monitor the conformational changes the docking complexes undergo over a stipulated time interval, the docking assemblies were subjected to molecular dynamics simulations for 200-ns time frame. All-atom additive protein force field, CHARMM36, available in the GROMACS 2021 package installed on a Linux-based system was utilized to perform the MD simulations (Abraham and Gready 2011; Huang and Mackerell 2013; Kutzner et al., 2019). The topology files of ligands were prepared using the ACPYPE (AnteChamber PYthon Parser interfacE) server (Sousa Da Silva and Vranken 2012). The docking complexes were contained in a triclinic simulation box and solvated with a TIP3P water model. Counter Na^+^ and Cl^−^ ions were added to neutralize the solvated system, followed by the quick energy minimization of the system with the help of the LINCS constraint algorithm and the steepest descent algorithm. The process of equilibrium was divided into two steps. In the first step, equilibration was established with a constant number of particles, volume, and temperature (NVT), with a 500-ps timestep, while the second step was completed with a constant number of particles, pressure, and temperature (NPT) with the ensemble at 300 K. The particle mesh Ewald (PME) method was used to calculate the long-range electrostatic interactions (Abraham and Gready 2011). Prior to the production run, different thermodynamics properties (pressure, density, potential energy, and temperature) of the system were carefully monitored to verify adequate equilibration. The v-rescale, Berendsen temperature coupling method was employed to regulate the temperature inside the box. After completion of the pressure and temperature equilibration of the system, a production run of 200 ns was run with each step of 2 fs

### Molecular Dynamics Trajectory Analysis

After the successful completion of the 200 ns MD simulation run, different factors of MD, namely, the RMSD (root mean square deviations), RMSF (root mean square fluctuations), number of hydrogen bonds, Rg (the radius of gyration), and SASA (solvent accessible surface area) were calculated using a set of tools embedded in the GROMACS package. The RMSD plot of all complexes was calculated using the gRMS tool, while the RMSF was generated using the gRMSF module of the GROMACS. The Rg, SASA, and hydrogen bonds were estimated using gyrate, gmxsasa, and g h bond tools, respectively.

### Binding Free Energy Calculations

After the MD simulations of the protein–ligand complexes were completed, the molecular mechanics/Poisson–Boltzmann surface area (MM/PBSA) binding free energy was calculated for all four docking complexes using the g_mmpbsa script program developed by Kumari et al. (2014). The g_mmpbsa module was embedded in the GROMACS package to integrate high-throughput MD simulation with calculations of binding free energy (Kumari et al., 2014; Aldeghi et al., 2017). The major components of energy, namely, binding energy (kJ/mol), van der Waal energy (∆EvdW), electrical energy (∆Elec), polar solvation energy (∆G polar), and solvent-accessible surface area (SASA) were calculated using the MM/PBSA method through the MD trajectories as described in previous reports (Kumar et al., 2020; Kumar et al., 2021; Mishra et al., 2021; Pathak et al., 2021).

In general, the following equation can be used to calculate the MM/PBSA method–based binding free energy of docking complexes:

$$\text{Δ}{\text{G}}_{\text{M}\text{M}\text{P}\text{B}\text{S}\text{A}}={\langle {\text{G}}_{\text{c}\text{o}\text{m}\text{p}\text{l}\text{e}\text{x}}-{\text{G}}_{\text{p}\text{r}\text{o}\text{t}\text{e}\text{i}\text{n}}-{\text{G}}_{\text{l}\text{i}\text{g}\text{a}\text{n}\text{d}}\rangle }_{\text{c}\text{o}\text{m}\text{p}\text{l}\text{e}\text{x}},$$

where G_protein_ and G_ligand_ denote the total free energies of the isolated protein and ligand in the solvent, and G_complex_ depicts the total free energy of the docking complex, respectively.

## Results and Discussion

### Molecular Docking

**FIGURE 2:**
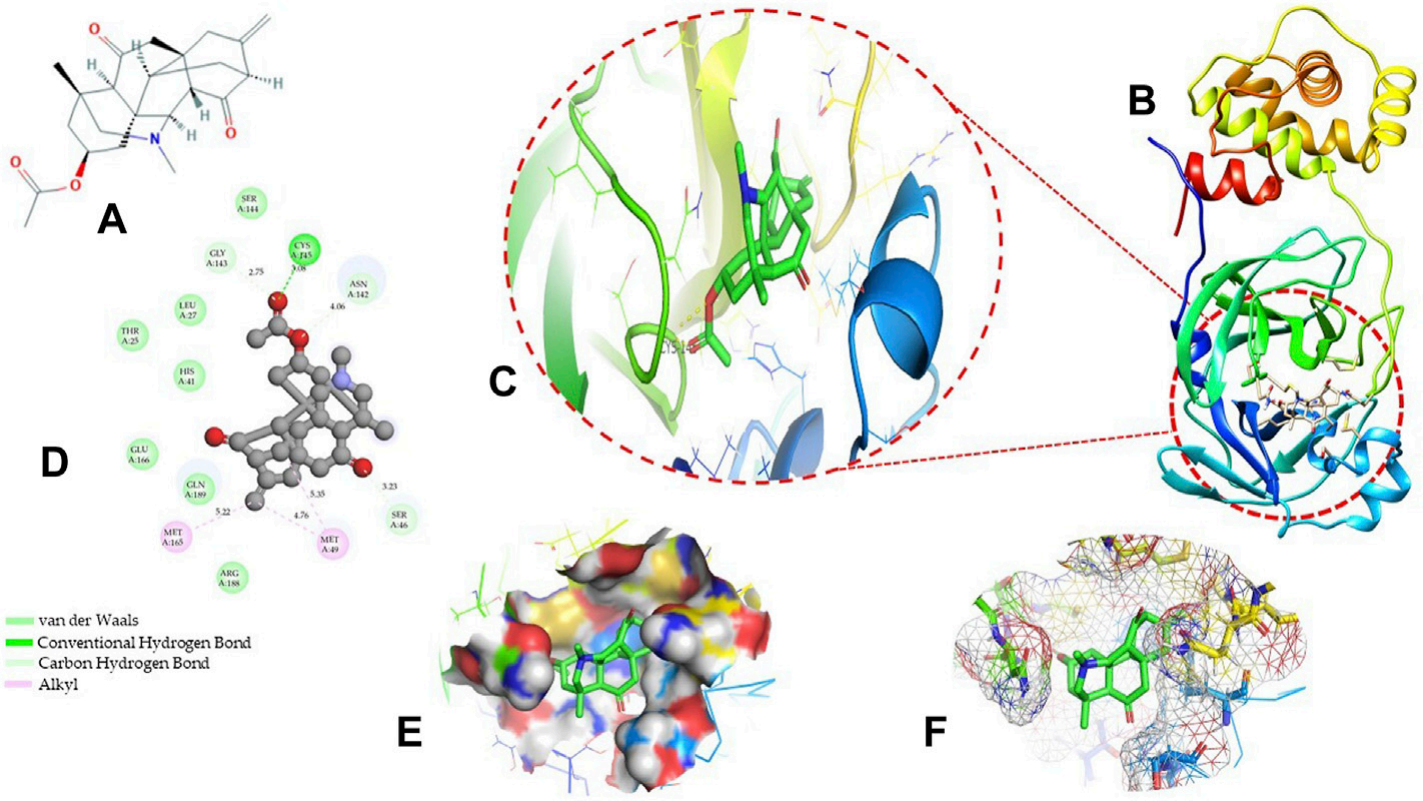
Representation of molecular interaction between M^pro^ and panicutine (CID_44566630) in 2D and 3D modes: **(A)** 2D structure of panicutine compound obtained from PubChem; **(B)** cartoon representation of the docking complex between M^pro^ and panicutine molecule; **(C)** a close view of the pocket with docked panicutine which is shown as green sticks, M^pro^ residues shown as atom-type color sticks, hydrogen bond represented as yellow dotted lines; **(D)** 2D plot of molecular interactions (van der Waals, conventional hydrogen bond, carbon–hydrogen bond, and alkyl) between panicutine and M^pro^; **(E)** solid surface representation of pocket site; and **(F)** mesh surface view of pocket with panicutine in green sticks. The docking complex is rendered in 2D and 3D shapes using different protein visualization tools such as Discovery Studio, UCSF Chimera, and PyMOL.

**FIGURE 3:**
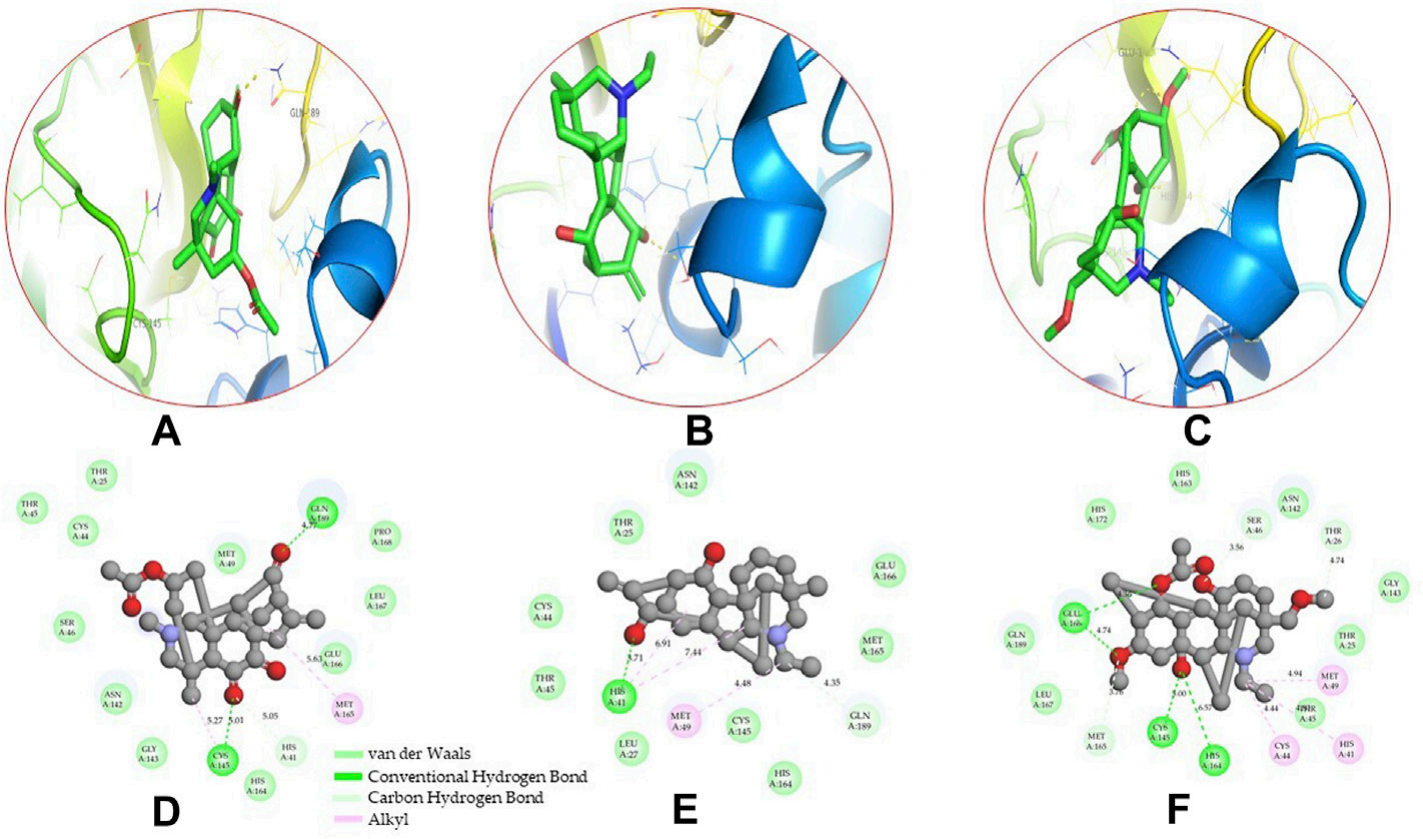
3D and 2D representations of the docking complexes between vilmorrianone, denudatine, condelphine, and M^pro^ of COVID-19; **(A)** 3D binding mode of vilmorrianone compound with M^pro^ active site; **(B)** 3D binding mode of denudatine compound with M^pro^ active site; **(C)** 3D binding mode of condelphine compound with M^pro^ active site. Molecules are represented as green sticks, M^pro^ residues depicted as atom-type color sticks, while yellow dotted lines show the hydrogen bonds between natural alkaloids and M^pro^ of COVID-19; **(D)** 2D representation of M^pro^ and vilmorrianone complex; **(E)** 2D representation of M^pro^ and denudatine complex; and **(F)** 2D representation of M^pro^ and condelphine complex. Molecular interactions such as van der Waals, conventional hydrogen bond, carbon–hydrogen bond, and alkyl are indicated by different colors. 3D and 2D representations of docking complexes are rendered with the help of PyMOL and Discovery Studio programs, respectively.

Molecular docking has become one of the most important molecular modeling methods to study the mechanism of interaction between enzymes and ligands and investigate how receptors and ligands fit together in a significant manner (Meng et al., 2011; Jee et al., 2017; Salmaso and Moro 2018). This revolutionary method has been utilized in the process of computer-aided drug design (CADD) to discover potential drug candidates against different diseases (Wadood et al., 2013; Yu and MacKerell 2016; Salmaso and Moro 2018). There is a critical need for effective drugs against COVID-19 since treatments are still limited. The screening of drug candidates against important targets of SARS-CoV-2 is a valid approach to solve this dilemma. In the present study, to investigate the inhibition potency and gain insight into the possible mechanisms of Jadwar-derived natural alkaloids: panicutine, vilmorrianone, denudatine, and condelphine, molecular docking was performed on M^pro^ of COVID-19. These compounds were docked with the M^pro^ binding pocket. These bioactive molecules have been found to have a binding energy of −7.4, −7.0, −6.0, and −5.9 kcal/mol, respectively. Out of these four docked compounds, the panicutine molecule ranked as the top interacting molecule with M^pro^ based on the calculated higher negative binding energy. Details of the chemical structures (2D and 3D), PubChem IDs, binding energy scores, hydrogen bonding, carbon–hydrogen (C-H) bonds, and alkyl interactions between the M^pro^ of COVID-19 and the four natural alkaloids are presented in Table 1. Two different programs, Discovery Studio and PyMOL, have been utilized to visualize the docking interactions in the form of 2D and 3D plots. As evident from Figure 2, the panicutine formed the hydrogen bond with Cys145 (3.08 Å) residue. Three residues namely, Ser46 (3.23 Å), Asn142 (4.06 Å), and Gly143 (2.75 Å), demonstrated the C-H bonds. Panicutine was found to have two alkyl bonds with Met49 (4.76, 4.77, 5.35 Å) and Met165 (5.22 Å) residues. Seven residues such as Thr25, Leu27, His41, Ser144, Glu166, Arg188, and Gln189 manifest van der Waals (VdW) interactions. In the vilmorrianone compound, two residues Cys145 (5.01 Å) and Gln189 (4.77 Å) exhibit hydrogen bond interactions. The residues His41 (5.05) and Met165 (5.63 Å) showed C-H bonds and alkyl interactions, respectively. Eleven residues, namely, Thr25, Cys44, Thr45, Ser46, Met49, Asn142, Gly143, His164, Glu166, Leu167, and Pro168, demonstrated VdW interactions (Figures 3A,D). As shown in Figures 3B and E, residue His41 (5.26 Å) formed a single hydrogen bond with the denudatine molecule. Residue Gln189 (4.35 Å) showed C-H bonds. Three residues, namely, Met49 (4.92 Å), Cys145 (3.71 Å), and Met165 (4.59 Å), manifest alkyl bonds. Seven residues, namely, Leu27, Thr25, Cys44, Thr45, Asn142, His164, and Glu166, interacted with the denudatine compound *via* VdW. In the case of condelphine, three residues, namely, Cys145 (5.00 Å), His164 (6.57 Å), and Glu166 (4.36 Å, 4.74 Å), formed hydrogen bonds. Three residues Thr26 (4.74 Å), Ser46 (3.56 Å), and Met165 (3.76 Å) interacted through C-H bonds. Two residues Cys44 (3.83 Å) and Met49 (4.58 Å) formed alkyl bonds. Condelphine also manifests VdW interaction with nine residues, namely, Thr25, Thr45, His41, Asn142, Gly143, His 163, Leu167, His172, and Gln189 (Figures 3C and F). We also predicted the contact maps of interactions between Jadwar-derived alkaloids and M^pro^ using the PDBsum web server to study the relative positions of the ligand molecules to the binding site of Mpro. As evident from Supplementary Figure S1, the predicted contact interaction maps show the hydrogen bond distances between 2.72 and 3.32 Å, while the nonbonded interaction shows a higher range of distances between the binding site and ligand molecules. The binding site residues which are not involved in forming the hydrogen bond with ligands also show a higher range of distances. Previously, different *in silico* and *in vitro* studies reported a similar trend of tightly fitting inhibitors in the binding pocket of SARS-CoV-2 M^pro^, which confirms our study (Park et al., 2015; Aanouz et al., 2020; Bello et al., 2020; Chikhale et al., 2020; Krupanidhi et al., 2020; Kumar et al., 2020; Muhammad et al., 2020; Tripathi et al., 2020; Mitra et al., 2021; Varadharajan et al., 2021; Ali et al., 2022; Linani et al., 2022).

**TABLE 1:** Details of molecular docking of Jadwar-derived alkaloids against the main protease of COVID-19.

Compound name	PubChem ID	Chemical structure		Binding energy (kcal/mol)	Molecular interactions
		2D	3D		
Panicutine	CID_44566630	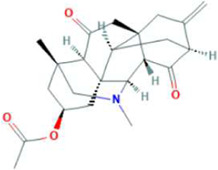	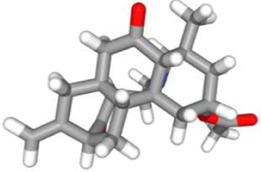	−7.4	Hydrogen bond: CYS145 (3.08 Å)
					Carbon-–hydrogen bond: SER46 (3.23 Å), ASN142 (4.06 Å), GLY143 (2.75 Å)
					Alkyl: MET49 (4.76, 4.77, 5.35 Å), MET165 (5.22 Å)
Vilmorrianone	CID_44566629	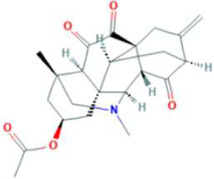	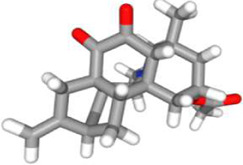	−7.0	Hydrogen bond: CYS145 (5.01 Å), GLN189 (4.77 Å)
					Carbon–hydrogen bond: HIS41 (5.05)
					Alkyl: MET165 (5.63 Å)
Denudatine	CID_161515	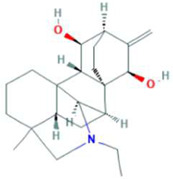	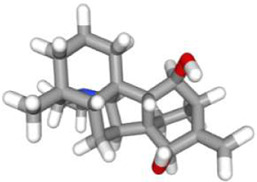	−6.0	Hydrogen bond: HIS41 (5.26 Å)
					Carbon–hydrogen bond: GLN189 (4.35 Å)
					Alkyl: MET49 (4.92 Å), CYS145 (3.71 Å), MET165 (4.59 Å)
Condelphine	CID_441720	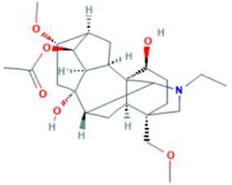	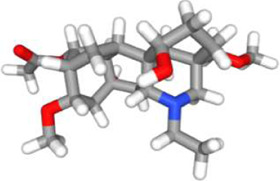	−5.9	Hydrogen bond: CYS145 (5.00 Å), HIS164 (6.57 Å), GLU166 (4.36 Å, 4.74 Å)
					Carbon–hydrogen bond: THR26 (4.74 Å), SER46 (3.56 Å), MET165 (3.76 Å)
					Alkyl: CYS44 (3.83 Å), MET49 (4.58 Å)

The interest to develop effective antiviral therapy against COVID-19 from the natural products of medicinal plants has increased globally. In several studies, phytochemicals were found to exhibit promising inhibitory effects against the M^pro^ of COVID-19 (Benarba and Pandiella 2020; Huang et al., 2020; Verma et al., 2020; Ahmad et al., 2021). Kumar et al. (2020) demonstrated that three natural bioactive molecules, namely, ursolic acid, carvacrol, and oleanolic acid, were able to inhibit M^pro^ protein in a significant manner. The binding affinity of these natural metabolites was found to be −5.9, −4.0, and −6.0 kcal/mol, respectively. In a recent study, Mishra et al. (2021) investigated the inhibition potencies of a set of natural compounds from medicinal plants as promising inhibitors against SARS-CoV-2 M^pro^. Based on the integrated molecular docking and modeling analysis, four phytochemicals, namely, amentoflavone, guggulsterone, puerarin, and piperine, were reported as top-ranked molecules. In a recent follow-up study, Kumar et al. (2021) explored sesame-derived natural compounds as antiviral molecules against M^pro^. The virtual screening of an in-house library which contains 36 natural sesame-derived compounds exhibited four bioactive molecules, namely, sesamin, sesaminol, sesamolin, and sesamolinol, as the top interacting compounds to the M^pro^ of COVID-19. Out of these four molecules, the sesamin compound showed a higher negative binding energy of −6.7 kcal/mol and formed three strong hydrogen bonds with Asn151 (5.46 Å), Ser158 (4.38 Å), and Arg298 (6.05 Å) residues. The interaction mechanisms of natural compounds against M^pro^ of COVID-19 reported in these studies are consistent with the docking results of the present study.

To date, several studies have been performed to investigate the inhibition potential of alkaloid compounds against different drug targets of SARS-CoV-2, namely, spike glycoprotein, nucleocapsid, angiotensin-converting enzyme 2 (ACE2), RdRp, and 3CL^pro^ (Garg and Roy 2020; El-Demerdash et al., 2021; Ghosh et al., 2021; Ismail et al., 2021; Majnooni et al., 2021). A docking study reported thalimonine and sophaline D as potential inhibitors against the M^pro^ of COVID-19 with binding energies of −8.39 and −8.79 kcal/mol, respectively (Garg and Roy 2020). In another study, a *Justicia adhatoda*–derived alkaloid compound, namely, anisotine, showed interaction with two catalytic residues (His41 and Cys145) of the M^pro^ with a binding score of −7.9 kcal/mol (Ghosh et al., 2021). Of note, two alkaloids (quinoline and quinazoline) have been previously shown to be effective against three-drug targets of COVID-19, namely, M^pro^, spike glycoprotein, and ACE2 (Ismail et al., 2021). In a recent study, El-Demerdash et al. (2021) performed the virtual screening approach to screen a library of alkaloids to identify the promising inhibitors of multidrug targets for SARS-CoV-19. Based on the docking affinity, pentacyclic alkaloids, crambescidin and crambescidin, have been proposed as the top interacting molecules for five drug targets of COVID-19, namely, the M^pro^, spike glycoprotein, nucleocapsid phosphoprotein, membrane glycoprotein, and a nonstructural protein (nsp10). Our docking results may support previous reports on the inhibition potential of natural alkaloids against the M^pro^ of COVID-19.

### Pharmacokinetics Evaluation

The promising docking results enabled us to explore the ADMET properties of compounds, therefore, pharmacokinetic characteristics of four Jadwar-derived natural alkaloids (panicutine, vilmorrianone, denudatine, and condelphine) were evaluated prior to conducting the MD analysis using automated SwissADME server. The calculated pharmacokinetic properties of these compounds are shown in Table 2. Panicutine, vilmorrianone, denudatine, and condelphine have the following molecular weights, respectively: 383.48, 397.46, 343.50, and 449.58 g/mol; these four natural alkaloids have a molecular weight ≤500 g/mol. The molecular weight characteristics of these molecules suggested that they can easily be transported, diffused, and absorbed in the body in a significant manner (Lipinski 2004). The LogP values of panicutine, vilmorrianone, denudatine, and condelphine compounds were found to be 2.32, 1.72, 2.85, and 1.71, respectively, which meet the essential conditions of the Lipinski’s rule of five. The calculated number of hydrogen bond donors of these four molecules was less than five which is in accordance with ADME as the number of H-bond donors must be ≤5. The pharmacokinetics analysis suggested that panicutine, vilmorrianone, denudatine, and condelphine alkaloids represent the following values of the topological polar surface (TPSA): 63.68, 80.75, 43.70, and 88.46 Å^2^. The lower TPSA values indicate the acceptable range of results and were found to be consistent with previous reports which showed the natural products as promising inhibitors of SARS-CoV-2 M^pro^ (Kumar et al., 2020; Kumar et al., 2021; Mishra et al., 2021). The Jadwar-derived natural alkaloids proposed in the present study also meet the essential criteria of Veber’s rule which defines the oral bioavailability of drug-like molecules. Panicutine, vilmorrianone, denudatine, and condelphine have molar refractivity values of 107.23, 107.43, 103.06, and 121.71, respectively; these alkaloids also present the synthetic accessibility (SA) scores of 5.78, 5.82, 5.91, and 6.17, respectively. In the drug designing process, SA has been considered as one of the essential parameters (Ertl and Schuffenhauer 2009). The calculated SA score of these molecules was found to be <10, which conforms with previous reports and reveals that these alkaloids can be synthesized easily (El-Demerdash et al., 2021; Ghosh et al., 2021; Kumar et al., 2021). Altogether, the pharmacokinetics evaluation suggested that these Jadwar-derived natural alkaloids harbor favorable drug-likeness properties and could be considered as therapeutic agents.

**TABLE 2:** Pharmacokinetics evaluation of natural alkaloids derived from Jadwar (*D. denudatum*).

Drug likeliness	MW (g/mol)	Consensus log Po/W (range ≤5)	No. of H-bond acceptors (range ≤10)	No. of H-bond donors (range ≤5)	Molar refractivity (range 40-130)	Lipinski	Veber	Bioavailability score (range 0.4–0.6)	Synthetic accessibility (range >6)	TPSA (Å2)	No of rotatable bonds (range 1–10)	Solubility (mg/ml)
Alkaloids	(range ≤500 g)									(range >100)		
Panicutine (CID_44566630)	383.48	2.32	5	0	107.23	Yes	Yes	0.55	5.78	63.68	2	7.14e-02 (Soluble)
Vilmorrianone (CID_44566629)	397.46	1.72	6	0	107.43	Yes	Yes	0.55	5.82	80.75	2	9.82e-02 (Soluble)
Denudatine (CID_161515)	343.50	2.85	3	2	103.06	Yes	Yes	0.55	5.91	43.70	1	4.05e-01 (Soluble)
Condelphine (CID_441720)	449.58	1.71	7	2	121.71	Yes	Yes	0.55	6.17	88.46	6	2.05e+00 (Soluble)

### Docking Complexes Showed Stability Throughout the Molecular Dynamics Simulations on 200 ns

Over the past years, MD simulations-based methods have expanded dramatically in the field of structural biology and drug discovery to design novel therapeutics against contagious diseases (Gajula et al., 2016; Hollingsworth and Dror, 2018). These revolutionary methods provide the ability to assess the stability and behavior of biological macromolecules and their molecular interactions with ligand molecules at very fine temporal resolution. In the present study, MD simulations were conducted for 200 ns using the docked conformation of M^pro^–panicutine, M^pro^–vilmorrianone, M^pro^–denudatine, and M^pro^–condelphine complexes to evaluate the stability and investigate the molecular interactions at the atomic level. The dynamic behavior of the simulated systems was analyzed using different functions, namely, RMSD, RMSF, hydrogen bond, Rg, and SASA.

### Root Mean Square Deviations

**FIGURE 4:**
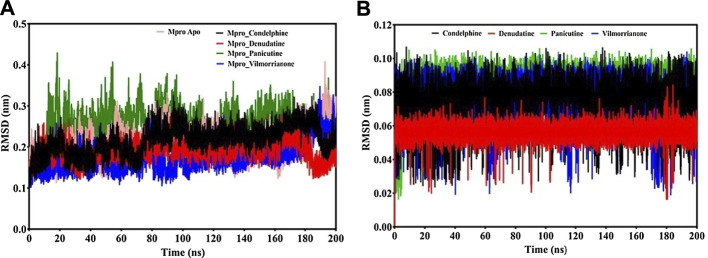
RMSD analysis: **(A)** backbone RMSD plot of docking complexes and **(B)** ligand RMSD plot of docking complexes [control (pink), M^pro^–condelphine (black), M^pro^–denudatine (red), M^pro^–panicutine (green), and M^pro^–vilmorrianone (blue)].

Calculating the RMSD plot is a well-established method to investigate the stability of docking complexes. All the ligand and backbone RMSDs were graphically studied to check the stability of the docking complexes. From the RMSD plot shown in Figure 4A, it can be observed that the M^pro^ backbone exhibited a constant range of stability throughout the simulation with a range between ∼0.1 and ∼0.43 nm. The average RMSD values for the M^pro^ complexes with panicutine, vilmorrianone, denudatine, and condelphine were 0.25, 0.17, 0.19, and 0.21 nm, respectively (Table 3). The M^pro^ Apo (pink) which has been considered as the control showed an average RMSD value of 0.2 nm. Along with the control, most of the docking complexes demonstrated small fluctuations between 20 and 80 ns. After 80 ns, the docking complexes showed stability up to 180 ns. As shown in Figure 4A, panicutine (green) showed the largest fluctuations between 20 and 100 ns; however, after 100 ns, panicutine (green) reflected stability on the 200-ns time scale around ∼0.3 nm. Condelphine (black) presented as the second most fluctuated; this molecule showed two fluctuations between 10–80 and 180–200 ns. Two complexes, vilmorrianone (blue) and denudatine (red), also exhibited small fluctuations between 185 and 200 ns; however, no conformational changes were noted in the receptor protein structure upon the binding of ligand molecules. As compared with the control, all four ligands presented similar patterns of stability and average RMSD with small conformational changes. As expected, the calculated ligand RMSD plot also showed the constant range of target molecules' stability with small fluctuations over time. The average ligand RMSD values of panicutine, vilmorrianone, denudatine, and condelphine were 0.08, 0.07, 0.05, and 0.07, respectively. As shown in Figure 4B, the measured ligand plot represents the stability of ligands with small fluctuations in condelphine (black), panicutine (green), and vilmorrianone (blue). Condelphine (black) showed small fluctuations at the initial point (5–40 ns) on 0.9 nm. At the starting point, panicutine (green) also depicted small fluctuations up to 10 ns. After 10 ns, the vilmorrianone (blue) showed two fluctuations, the first between 10 and 20 ns on ∼0.9 nm and the second between 175 and 185 ns on ∼ 0.5 nm. Denudatine (red) reflected the straight line without notable fluctuations throughout the simulations on 200 ns. Therefore, the binding of denudatine made the complex more stable. Based on the analysis of the protein backbone and ligand RMSD plots, it can be concluded that the measured RMSDs demonstrated minimally and the docking complexes were stable with significant potential to compare with structures available in the structure repositories.

**TABLE 3:** Calculated values of different components of MD simulations such as protein backbone RMSD, ligand RMSD, RMSF, Rg, SASA, and H-bonds.

Components	Apo	Condelphine	Denudatine	Vilmorrianone	Panicutine
RMSD protein (nm)	0.2	0.21	0.19	0.17	0.25
RMSD ligand (nm)	-	0.07	0.05	0.07	0.08
RMSF (nm)	0.19	0.1	0.1	0.11	0.14
Rg (nm)	2.24	2.2	2.23	2.23	2.25
SASA (nm^2^)	153.41	147.57	150.46	150.22	153.96
H-bonds (#)	-	2	1	1	1

### Root Mean Square Fluctuations

**FIGURE 5:**
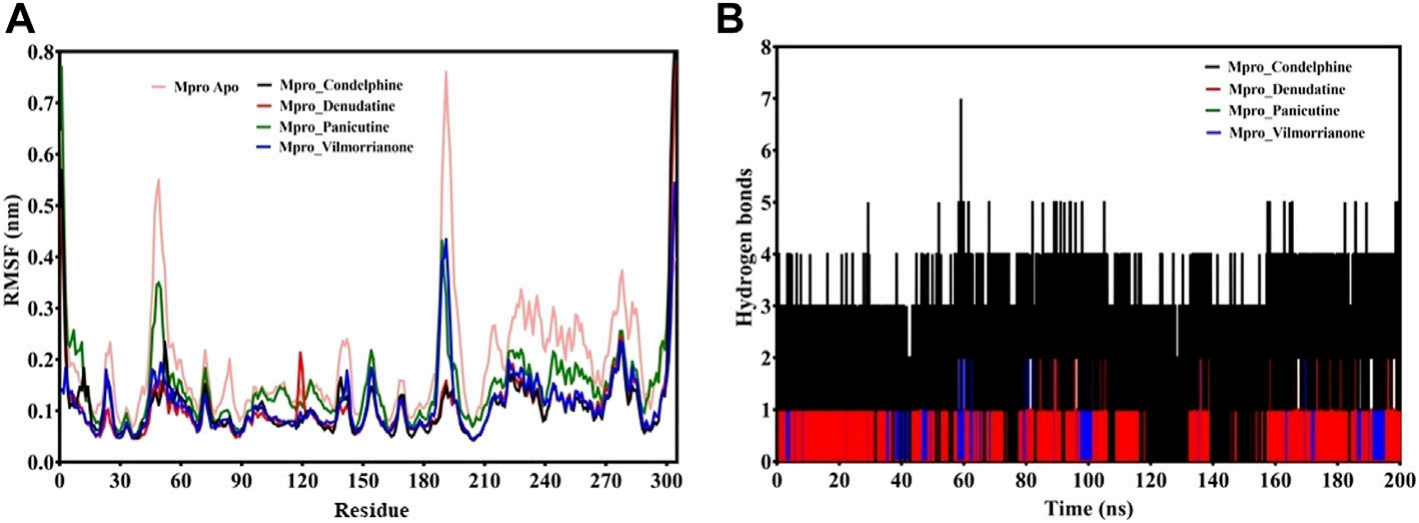
**(A)** RMSF plot of docking complexes and **(B)** distribution of hydrogen bonds [control (pink), M^pro^–condelphine (black), M^pro^–denudatine (red), M^pro^–panicutine (green), and M^pro^–vilmorrianone (blue)].

The RMSF plot analysis was conducted with the primary goal to assess the mobility of residues upon binding of the ligand molecules. Per the general phenomena of the RMSF analysis, a high fluctuations score depicts more flexibility and unstable bonds, while a low value represents the correct structure regions in the docking complexes (Martínez 2015; Gajula et al., 2016). The RMSFs of the alpha carbon atoms of all simulated systems were investigated in the present study and are shown in Figure 5A. All five simulated systems, namely, the control, M^pro^–panicutine, M^pro^–vilmorrianone, M^pro^–denudatine, and M^pro^–condelphine, demonstrated a close pattern of fluctuations throughout the simulation on a 200-ns time scale. The average RMSF values of the control, M^pro^–panicutine, M^pro^–vilmorrianone, M^pro^–denudatine, and M^pro^–condelphine docking complexes were 0.19, 0.14, 0.11, 0.1, and 0.1 nm, respectively. These values clearly reflect that all docking complexes show relatively less conformation fluctuations than the control system. It is observed from the RMSF plot on Figure 5A that two complexes, M^pro^–panicutine (green) and M^pro^–vilmorrianone (blue) show the highest peak between 180 and 200 residues on 0.4 nm. The fewer fluctuations noted in the protein–ligand complexes support the docking findings and reveal that the M^pro^ significantly interacts with panicutine, vilmorrianone, denudatine, and condelphine compounds.

### Hydrogen Bond Analysis

To determine the strength of binding of panicutine, vilmorrianone, denudatine, and condelphine to the target M^pro^, the number of intermolecular hydrogen bonds was calculated by utilizing the MD trajectories. Panicutine (green), vilmorrianone (blue), and denudatine (red) formed one hydrogen bond with M^pro^, while condelphine (black) manifested two hydrogen bonds with the target receptor throughout the MD simulation on the scale of 200 ns. These results were the confirmation of the hydrogen bond interactions predicted using the molecular docking approach. Figure 5B represents the distribution of hydrogen bonds. The ligand molecules had a constant range of hydrogen bonds between one and two during the whole simulation. The results of the hydrogen bond analysis revealed that the intermolecular hydrogen bonds were stable, and the natural alkaloids considered in the present study could maintain a strong molecular interaction with the active site of the M^pro^ in a significant manner.

### Radius of Gyration and Solvent Accessible Surface Area Analysis

**FIGURE 6:**
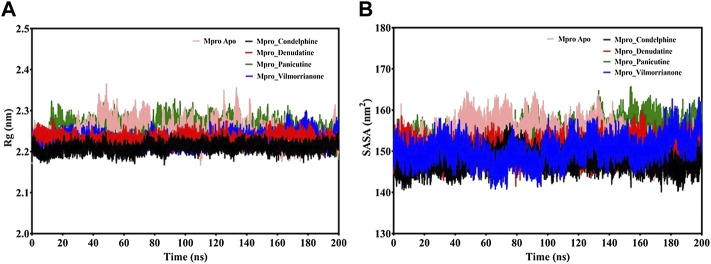
**(A)** Rg plot of docking complexes and **(B)** SASA plot of docking complexes [control (pink), M^pro^–condelphine (black), M^pro^–denudatine (red), M^pro^–panicutine (green), and M^pro^–vilmorrianone (blue)].

MD trajectories corresponding to the four docking complexes (M^pro^–panicutine, M^pro^–vilmorrianone, M^pro^–denudatine, and M^pro^–condelphine) were further evaluated with the help of integrated Rg and SASA analyses. The Rg plot analysis was calculated to extract the compactness of the simulated systems with the time scale. As evident from Figure 6A, the Rg values of the control and docking complexes are noted between ∼2.2 and ∼2.3 nm during the simulation on 200 ns. The average Rg values of the control, M^pro^–panicutine, M^pro^–vilmorrianone, M^pro^–denudatine, and M^pro^–condelphine docking complexes are 2.24, 2.25, 2.23, 2.23, and 2.2 nm, respectively. These Rg values demonstrate that all the protein–ligand complexes except M^pro^–panicutine (green) showed relatively less value than the control. The calculated Rg values for these alkaloid compounds are in consent with previous findings which have reported that the medicinal plants–derived bioactive molecules are potential inhibitors against M^pro^ of COVID-19 (Kumar et al., 2020; Mishra et al., 2021). In the present study, the calculated Rg values confirm the stability of every docking complex and reflect that the binding of the natural alkaloids does not induce structural changes throughout the simulation on a 200-ns time scale.

The analysis of the SASA plot is an important step toward the investigation of the receptor exposed to solvents during the MD simulations on different nanoseconds. Per the general rule, a higher SASA value indicates the expansion of protein volume. During MD simulations, low fluctuations for the docking complexes are always considered good. The calculated SASA plot for the control and docking complexes of the natural alkaloids and M^pro^ is given in Figure 6B. The SASA values are noted between 145 and 160 nm^2^. The average Rg values of the control, M^pro^–panicutine, M^pro^–vilmorrianone, M^pro^–denudatine, and M^pro^–condelphine docking complexes are 153.41, 153.96, 150.22¸150.46, and 147.57 nm^2^, respectively. All the complexes showed the SASA values as less than those of the control system except for the M^pro^–panicutine complex. The outcome of the SASA analysis suggests the stability of the docking complexes and also indicates that binding of panicutine, vilmorrianone, denudatine, and condelphine does not affect the protein folding.

### Calculation of Binding Free Energy

To achieve accurate binding free energy estimation of the protein ligand complexes (M^pro^–panicutine, M^pro^–vilmorrianone, M^pro^–denudatine, and M^pro^–condelphine), we employed the MmPbSaStat.py python script embedded in the g_mmpbsa module. The MM/PBSA method is a widely used accurate method to calculate the ligand-binding affinities (Hou et al., 2011; Genheden and Ryde 2015). The scores of the calculated binding free energy (van der Waal energy, electrical energy, polar solvation energy, and SASA) are provided in Table 4. As reported in previous reports, the final binding energy of the protein–ligand complex is represented by the cumulative sum of the different energies (van der Walls, electrostatic, polar solvation, and SASA) (Jee et al., 2017; Pathak et al., 2021). In the present study, all types of energies such as van der Walls, electrostatic, polar solvation, and SASA contributed to molecular interactions between the alkaloids and M^pro^ of COVID-19 in a significant manner. The calculated binding free energy of the Jadwar-derived alkaloids is as follows: panicutine, −140.758 ± 23.707 kJ/mol; vilmorrianone, −147.091 ± 10.059 kJ/mol; denudatine, −138 ± 13.873 kJ/mol; and condelphine, −127.939000 ± 13.931 kJ/mol. As a general fact, more negative values of the free binding energy depicted a stronger molecular interaction and increased affinity between the receptor protein and ligand molecules. Vilmorrianone (−147.091 ± 10.059 kJ/mol) possesses the maximum negative binding energy when compared with the other natural alkaloids considered in the present study, while panicutine (−140.758 ± 23.707 kJ/mol) exhibited the second least binding energy based on the MM/PBSA method of estimation. The complex of M^pro^–vilmorrianone showed lower binding free energy because of its stable interactions with binding site residues of M^pro^ at the atomic level. As evident from the MD simulation results, vilmorrianone also showed RMSD behavior in an acceptable range and displayed stability with small fluctuations throughout the MD simulation with an average RMSD value of 0.07 nm. Taken together, these natural alkaloids with maximum negative energy support the concept of design and validate the CADD approach; they also demonstrate and assure the inhibition potential of Jadwar-derived alkaloids against the M^pro^ of COVID-19.

**TABLE 4:** Calculated free binding energy of docking complexes of M^pro^ and natural alkaloids (panicutine, vilmorrianone, denudatine, and condelphine).

Complex	Binding energy (kJ/mol)	van der Waal energy (∆EvdW) (kJ/mol)	Electrical energy (∆Elec) (kJ/mol)	Polar solvation energy (∆G polar) (kJ/mol)	SASA energy (kJ/mol)
Panicutine	−140.758 ± 23.707	−168.672 ± 19.599	−29.311 ± 13.245	71.015 ± 10.117	−13.790 ± 1.331
Vilmorrianone	−147.091 ± 10.059	−192.801 ± 7.972	−14.844 ± 7.307	78.543 ± 9.421	−14.988 ± 0.868
Denudatine	−138 ± 13.873	−160.823 ± 11.989	−34.509 ± 11.582	70.585 ± 9.442	−13.811 ± 0.802
Condelphine	−127.939000 ± 13.931	−155.616 ± 12.975	−61.506 ± 8.677	104.370 ± 9.388	−15.187 ± 1.198

## Conclusion

The M^pro^ of SARS-CoV-2 is a well-validated drug target due to its principal role in viral replication. The screening of phytochemicals against this important drug target has become a promising strategy in the design of potential drug candidates using the CADD approach. In the present study, the inhibitory potential of four natural Jadwar-derived alkaloids, namely, panicutine, vilmorrianone, denudatine, and condelphine, that targeted M^pro^ was investigated using the integrated molecular docking and modeling methods. Based on the docking results, we demonstrated that all four bioactive molecules significantly bind and stably interact with the active site of M^pro^. Furthermore, MD simulations analysis was performed over 200 ns to evaluate the binding position and structural stability of the docking complexes using different components from the MD trajectories. The calculation of the binding free energy supported the MD simulation in a significant manner and confirmed their stability at the atomic level. Furthermore, Lipinski’s rule of five and ADME properties–based validation of these natural compounds suggested positive drug-likeness properties, which is an essential step toward demonstrating drug safety. The inhibition potential of these Jadwar-derived natural alkaloids against M^pro^ can also be validated in the wet-lab setting with the aid of cell culture and small animal experiments. Based on the molecular modeling investigations, the current study suggests that panicutine, vilmorrianone, denudatine, and condelphine have the potential to inhibit the M^pro^ of COVID-19 and, in the future, may be candidates for anti-viral therapy.

## Data Availability

The original contributions presented in the study are included in the article/Supplementary Material, further inquiries can be directed to the corresponding author.
