# Supplementary material for: Potential of Natural Alkaloids From Jadwar (Delphinium denudatum) as Inhibitors Against Main Protease of COVID-19: A Molecular Modeling Approach
Source: Front Mol Biosci. 2022 May 10;9:898874. doi: 10.3389/fmolb.2022.898874 (PMC9127362; doi:10.3389/fmolb.2022.898874)
Supplement: Supplementary file 1 [file Table1.DOCX]

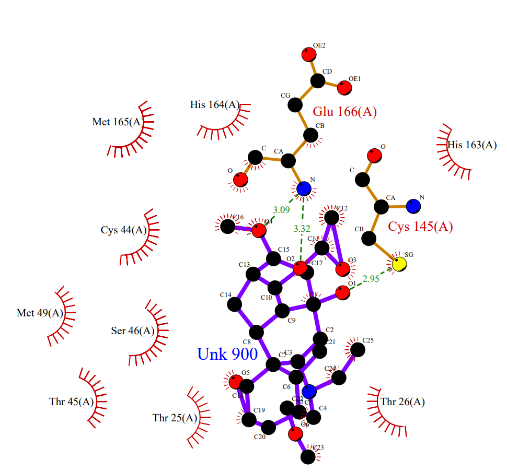

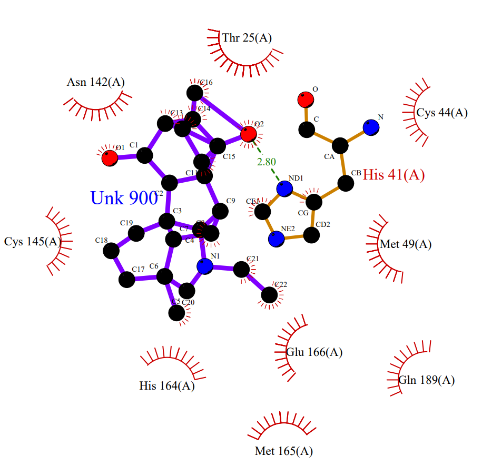


1. (b)


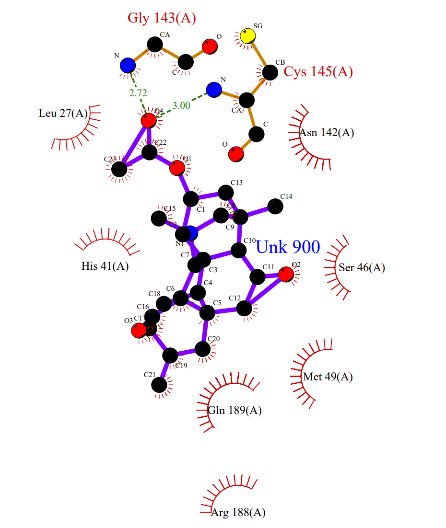

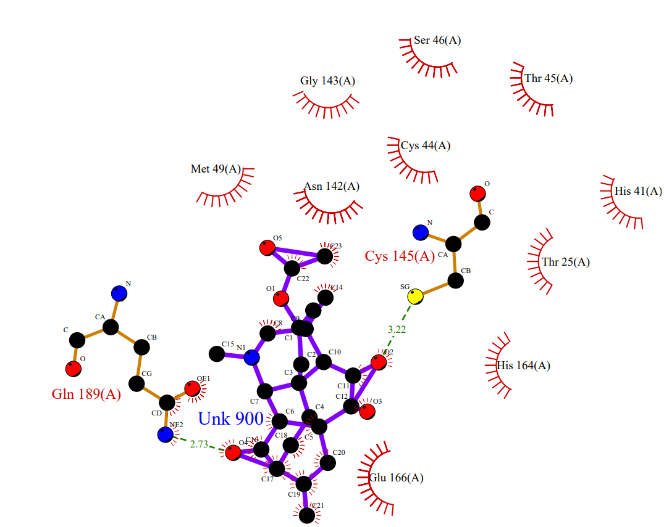


(c) (d)

**Suppl Figure 1**: Predicted contact maps between the M^pro^ protein and ligands: **(a)** M^pro^- condelphine; (**b**) M^pro^- denudatine; (**c**) M^pro^- panicutine; and (**d**) M^pro^- vilmorrianone. Contact maps predicted using Protein Database Summaries (PDBsum) web server of EBI (<http://www.ebi.ac.uk/thornton-srv/databases/pdbsum/Generate.html>)
